# Construction motion data library: an integrated motion dataset for on-site activity recognition

**DOI:** 10.1038/s41597-022-01841-1

**Published:** 2022-11-26

**Authors:** Yuanyuan Tian, Heng Li, Hongzhi Cui, Jiayu Chen

**Affiliations:** 1 Department of Architecture and Civil Engineering, City University of Hong Kong, Hong Kong SAR, China; 2 Department of Building & Real Estate, The Hong Kong Polytechnic University, Hong Kong SAR, China; 3 College of Civil and Transportation Engineering, Shenzhen University, Shenzhen, China; 4 School of Civil Engineering, Tsinghua University, Beijing, China

**Keywords:** Civil engineering, Occupational health

## Abstract

Identifying workers’ activities is crucial for ensuring the safety and productivity of the human workforce on construction sites. Many studies implement vision-based or inertial-based sensors to construct 3D human skeletons for automated postures and activity recognition. Researchers have developed enormous and heterogeneous datasets for generic motion and artificially intelligent models based on these datasets. However, the construction-related motion dataset and labels should be specifically designed, as construction workers are often exposed to awkward postures and intensive physical tasks. This study developed a small construction-related activity dataset with an in-lab experiment and implemented the datasets to manually label a large-scale construction motion data library (CML) for activity recognition. The developed CML dataset contains 225 types of activities and 146,480 samples; among them, 60 types of activities and 61,275 samples are highly related to construction activities. To verify the dataset, five widely applied deep learning algorithms were adopted to examine the dataset, and the usability, quality, and sufficiency were reported. The average accuracy of models without tunning can reach 74.62% to 83.92%.

## Abstract (summary)

**Table:** 

Measurement(s)	motion of construction workers
Technology Type(s)	wearable motion sensing system
Factor Type(s)	skeleton joints position
Sample Characteristic - Organism	construction workers
Sample Characteristic - Environment	construction site

## Background & Summary

Monitoring workers’ activities is essential for ensuring safety and tracking productivity of construction projects, as nearly 80% of fatal and nonfatal injuries are caused by unsafe behaviors on site^1^. In addition, activities associated with awkward postures, repetitive motions, and forceful exertions have imperceptible but grievous outcomes to construction workers’ long-term health, such as work-related musculoskeletal disorders (WMSDs)^2,3^. Conventional behavior-based safety management approaches^4^ rely on self-report, manual observation, and direct-measurement to identify unsafe behaviors^5–7^. Similar approaches are also popular for the assessment of labor productivity^8^. Due to its high time and effort cost^9^, automated and computational solutions with low-cost and easy-to-use sensors have been proposed by researchers. Human activities can be represented as sequences of 3D skeleton models, which usually can be constructed from motion capturing datasets. With deep learning and trajectory-based methods^10,11^, the sensing outputs, such as RBG videos, RGB-depth (RGBD) videos, and inertial signals, can be translated into human postures and activities^12^. These methods have been successfully implemented in various industries, such as healthcare^13^, sports^14^, gaming^15^, and cooking^16^. For the construction industry, researchers also proposed several models for RGB vision-based activity classification^17^ and inertial measurement units (IMU) based fall detection^18^.

Vision-based construction activity recognition is available and affordable. Researchers mainly rely on RGB and RGBD cameras. For example, Yang *et al*.^19^ utilized RGB video to classify 11 common construction activities. Roberts *et al*.^20^ used 317 annotated videos to analyze bricklaying and plastering worker’s operations. Khosrowpour *et al*. proposed a supervised machine approach to predict worker activities with RGB-D cameras and reported a recognition accuracy of 76%^21^. Another popular technical path is using wearable sensing systems, such as IMU sensors^18,22,23^, smartphones^24,25^, sports watches^26,27^, and wearable insole pressure systems^28–30^. For example, Yang *et al*.^18^ developed a semi-supervised learning algorithm to detect the near-miss falls of ironworkers with IMU systems. Antwi-Afari *et al*.^31^ assessed three types of recurrent neural networks for automated recognition and classification of construction workers’ awkward working postures using wearable insole sensors.

Given the importance and usefulness of these activity recognition algorithms, researchers have developed enormous and reliable datasets to support further theoretical development, such as the HDM05 Motion Capture (mocap) Dataset^32^, and the Berkeley MHAD Dataset^33^, the NTU + RGBD 120 Dataset^34^, and the CAD60 Dataset^35^. However, most of these datasets were designed for generic activity recognition but not specially designed for construction activities, resulting in high recognition errors and incorrect interpretations. This can be attributed to two reasons: first, exposure to awkward postures. Construction tasks involve complicated activities and heavily rely on manual operation. The physically demanding tasks make most construction workers suffer from work-related musculoskeletal disorders (WMSDs)^36^ and long-term ergonomic injuries. These injuries and WMSD are often attributed to awkward work postures^37^. Awkward posture refers to body postures that deviate significantly from normal and comfortable positions and can potentially lead to muscle disorders and trauma^38^. Awkward postures in construction activities are often associated with long-term muscle force exposure and body joint rotations near extreme due to various working tasks, such as load carrying, kneeling, bending, squatting, and twisting. The second reason is unique motion labels. The skeleton joint positions of many construction-related working activities in the context of the construction environment are often similar to generic operations of completely different tasks. For example, the skeleton posture of waving in daily life is identical to the motion of a construction worker painting drywall. Therefore, labels should be properly and specifically assigned. Due to the cost and time of constructing a new dataset, researchers tend to develop generic datasets for validating generic algorithms but neglect the unique data features and patterns of a specific industry. However, professional and specially designed datasets provide higher relevancy, accuracy, efficiency, and reliability for specific implementation^39^. To fill this gap, this study aims to develop a motion data library that is suitable for the development of activity recognition and task management in the construction industry by integrating a small, manually collected construction motion dataset with large-scale public datasets, aligning all datasets as one suitable, united, and properly labelled dataset.

## Methods

### Workflow of dataset development

**Fig. 1:**
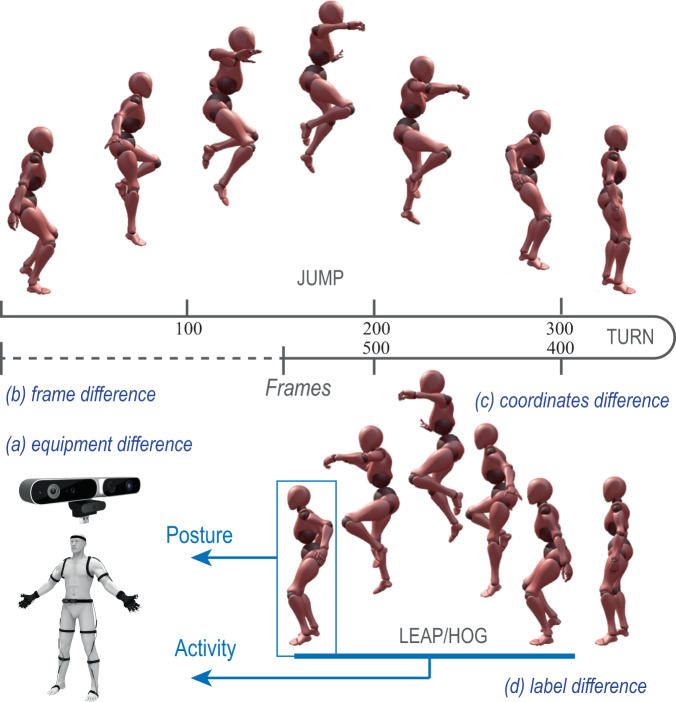
Inconsistencies in MoCap datasets.

To develop a construction motion dataset, a large-scale experiment to capture the major construction activities is necessary. However, given that many research teams have developed abundant generic motion datasets, this study combines both the existing datasets with the in-lab experiment dataset to compile an integrated construction motion dataset with specifically designed label systems. Doing so can translate generic motions to relevant construction activities through selection and annotation. This approach avoids repetitive motion capturing experiments, enlarges the construction motion dataset, and saves a significant amount of time and effort. However, combining existing public datasets and construction-specific datasets into one single integrated dataset has four major challenges as shown in Fig. 1. *(a)* Equipment Difference. Human motion can be captured through two streams of technologies: RGB/RGBD-based video processing and IMU-based wearable sensing. The choice of technology will result in differences in the final data format (as pictures or inertial signals) and data sampling rate. *(b)* Frame Difference. Different individuals can conduct the same activities with different durations. Also, due to the variation in sampling rates, even the same activity, and same duration can result in a different number of frames. *(c)* Coordinates Difference. With different equipment setups and coordination systems, the same activity can be represented differently by local or global coordination systems with different quantitative values. *(d)* Label Difference. The label used for the same activity can be different in different datasets, for example, “jump” also can be labelled as “leap” or “hop.”

Apart from aligning all public datasets in the same data format with the same skeleton model, this study also conducted an in-lab experiment to capture the predefined construction-related activities for further data annotation. The guidelines for study procedures complied with all relevant ethical regulations and approved by Human Subjects Ethics Committee of City University of Hong Kong. The informed consent was obtained from all participants. Before the in-lab experiment, 60 types of construction-related activities were predefined based on ergonomics analysis. The captured results were used as the standard posture sequences with other activity frames. The variations of joint movements of the skeleton models were computed. Then public datasets’ samples will be compared with standardized activities, and the label with the lowest variation or differences will be used to annotate the sample. The in-lab experiment utilized the Noitom Perception Neuron motion capture system and 10 subjects participated in the process of data collection.

**Fig. 2:**
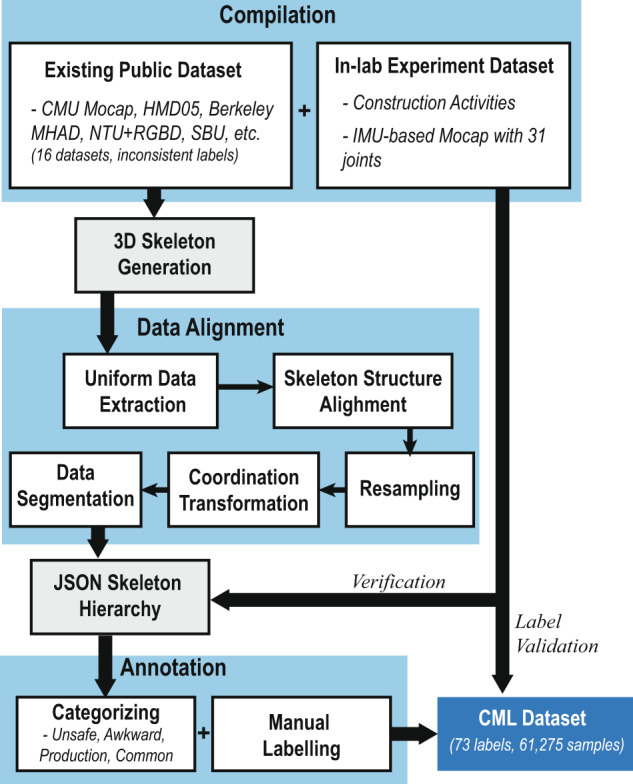
The workflow of the construction motion dataset development.

In summary, this study developed a formal workflow to process the manually collected in-lab experiment dataset and the public datasets (as shown in Fig. 2). To ensure the data format is consistent, all images, video frames, and inertial serial signals will be converted to 3D body skeletons. All skeleton data will be processed in four major steps, including uniform data extraction, skeleton structure alignment, resampling, and coordination transformation. Then all the aligned skeleton data will be manually annotated into four activity categories and assigned with labels. The final Construction Motion Library (CML) Dataset will be verified using the in-lab benchmarking data and tested with five popular deep learning algorithms. Table 1 summarizes the public skeleton motion datasets that were used in the study for the development of the construction motion dataset. Some public motion datasets focus on specific industries and have low relevancy to this study, so they were excluded from the study. For example, the Hollywood 3D Dataset^40^ was designed for performance activities.

**Table 1:** Public Datasets of Generic Motions Used in This Study.

	Dataset Name	File Format	Frame Rate	Size of Datasets			
				Sample	Subjects	Classes	Joints
0	In-lab Experiment	.bvh	120 Hz	--	10	60	31
		.raw					
1	CMU Mocap^72^	.asf	120 Hz	2,605	144	172	31
		.amc					
2	HMD05 Mocap^32^	.asf	120 Hz	2,286	5	26	31
		.amc					
3	Berkeley MHAD^33^	.bvh	480 Hz	671	12	11	30
4	NTU+RGBD 120^34^	.txt	30 Hz	114,480	40	120	25
5	SBU Kinect Interaction Dataset^73^	.txt	30 Hz	248	7	8	15
6	MSR Action3D Dataset^74^	.txt	15 Hz	567	10	20	20
7	CAD60 Dataset^35^	.txt	30 Hz	64	4	12	15
8	UTKinect-Action3D Dataset^75^	.txt	15 Hz	200	10	10	20
9	UCF Dataset^76^	.ske	30 Hz	1,280	16	16	15
10	Microsoft Research Cambridge-12^77^	.csv	30 Hz	594	30	12	20
11	Human 3.6M^78^	json	30 Hz	210	11	15	17
12	PKU_MMD^79^	.txt	30 Hz	20,780	66	51	25
13	SYSU 3D^80^	.txt	30 Hz	480	40	12	20
14	UTD Multimodal Human Action Dataset (UTD MHAD1)^81^	.mat	30 Hz	300	6	10	25
15	UTD MHAD2^81^	.mat	30 Hz	900	5	6	25
16	UTD MHAD3^81^	.mat	30 Hz	861	8	27	20

### Dataset alignment

To overcome the four technical challenges, this study developed a processing protocol for all datasets. The protocol has six major steps: *(1)* Skeleton Generation and Uniform Data Extraction. This step aims to design a uniform skeleton model and clean up the original datasets. *(2)* Skeletal Structure Alignment. This step ensures all the datasets using the same skeleton model and all the joints’ movements will be aligned in the same setting. *(3)* Resampling. This study aims to ensure all data sample follows the same timestamp system and have the same number of frames in unit time. *(4)* Coordination Transformation. This step ensures all samples have the sample local coordinate system and the same postures have the same quantitative values. *(5)* Data Segmentation. This step ensures the activity with the same label has the same frame length, which is easier for future usage. *(6)* Data Format of the CML Dataset. All the samples will be saved in the same data format for easier processing. The following paragraphs have a more detailed explanation of each step.

#### Skeleton generation and uniform data extraction

To align all datasets, both vision-based and IMU-based data formats were translated as 3D skeleton postures. A typical skeleton posture’s data form is in the Bio-Vision Hierarchy (BVH) format. The BVH data format can store both the joint connectivity and joint motions in a single file. As a widely used data format, all major motion files can be extracted as BVH motion files. Existing datasets usually store activity as separate files. However, many files have different enclosures, resulting in many labelled files having more than one activity and only having a rough tag. Therefore, to make all extracted files uniform, all data files with multiple activities were manually divided into short and independent activities and saved separately with a single label. Also, irrelevant motion frames were removed for clarity. For example, the original CMU Mocap Dataset has 2,605 activity sequences, and after data extraction and cleaning, the processed dataset has 172 activity types and 2,928 samples. Another issue is many activity files have multiple human subjects and null values. Null value samples are motion files whose skeleton joints’ motion is filled with zeros or partly missing some content. For example, in the UT-Kinect-Action3D Dataset, the “carry” activity has one sample full of zeros. In the NTU-RGB + D 120 Dataset, the activities of A1-A60 have 165 files filled with zeros, and the activities of A61-A120 have 191 files full of zeros. This study directly removed both the null value samples and samples with multiple human objects.

#### Skeletal structure alignment

**Fig. 3:**
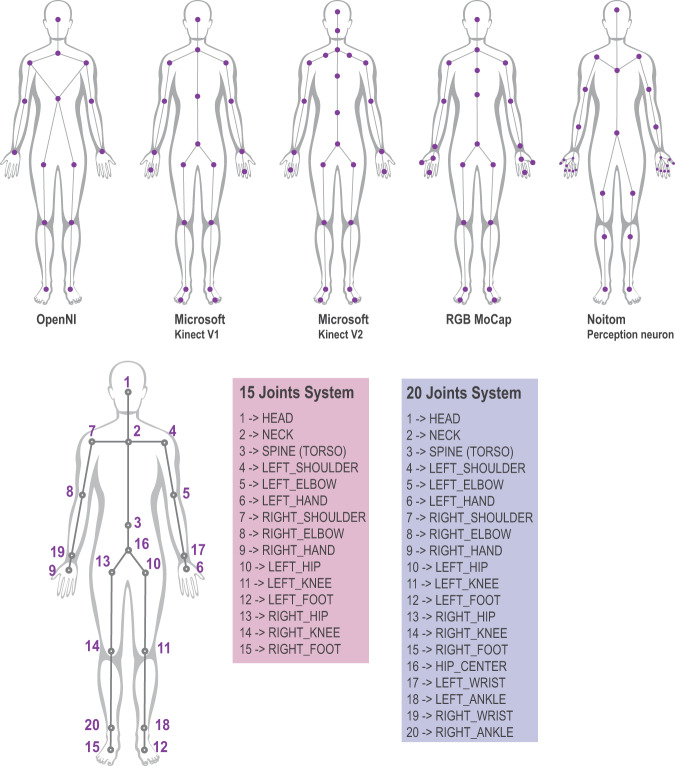
Typical skeletal body joints models and the simplified 15/20 joints system.

Prevalent motion capturing devices have their unique skeleton structures and technical configurations. As a result, the same motion may have different data structures in different datasets because of capturing devices, such as OpenNI (15 joints), Microsoft Kinect V1 (20 joints), Microsoft Kinect V12 (25 joints), RGB Mocap (17 joints), and Noitom Perception Neuron (29 + 2 joints). Figure 3 shows joint structures captured by five typical devices and the simplified 15/20-joint system for skeleton alignment. This study developed a 15–20 skeletons system, which can accommodate and be compatible with all other skeletal models. The system provides two joint structures, and the 20-joint model has more joints to quantify the motion with high resolution. The motion data of the same joints of the developed system and other skeleton models will remain the same. The missing joint will be computed with neighbor joint interpolation. The nonlinear interpolation utilized the multi-layer perceptron (MLP) model of the scikit-learn package (the validation error is 0.0961).

#### Resampling

The sampling rate of existing datasets ranges between 15 to 480 frames per second (fps). This results in high inconsistency in reading the proper inputs for posture recognition. For example, the Berkeley MHAD Dataset has a sampling rate of 480 fps, and the UTKinect-Action3D Dataset and MSR Action3D Dataset have a sampling rate of 15 fps.

This study converts all samples to 30 fps, which is the sampling rate of most devices (such as Kinect v1/v2 and Vicon). For datasets that have a sampling rate higher than 30 Hz, the redundant frames will be truncated; for datasets that have a sampling rate lower than 30 Hz, the missing frames’ data will be filled with the interpolated average results. In addition, as the datasets used different units, such as inch, centimeter, millimeter, and meter, all samples’ units will be converted into meters. In addition, some datasets have an z-axis calibration value to reflect the differences in subjects’ heights (such as the CMU Mocap Dataset having a scale length of 0.45 and the SBU Dataset having a z scaler of 7.8125). In this study, all samples’ z values will be adjusted according to their scaler.

#### Coordination transformation

**Fig. 4:**
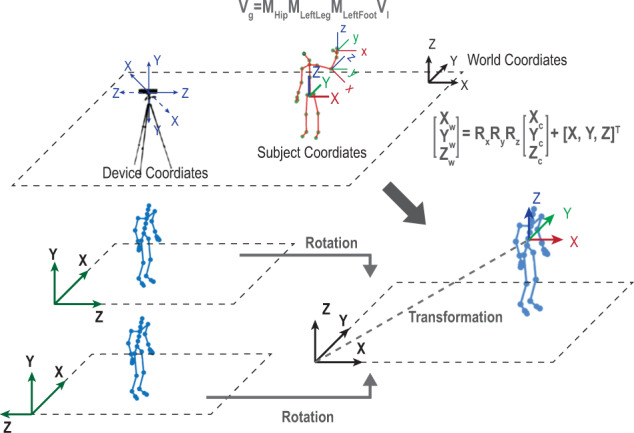
Conversion of coordination systems.

Different file formats not only result in various skeleton models but also introduce inconsistency in the coordination systems. The conversion of coordination systems needs to define proper rotation matrices (${R}_{x},{R}_{y},{R}_{z}$) and translation matrices (*T*). The skeleton-based motion files, such as .ASF/AMC and .BVH, define the recorded motion signals as a local system. Thus, to align the systems, each joint needs to be translated to the global system. Usually, all joints will be translated to the system at the hip joint through the multiplication of connected joints’ transformation matrices (*M*_*i*_). *M*_*i*_ can be computed with a relevant rotation matrix and translation matrix. Figure 4 shows such a transformation process for different coordination systems. As shown in the figure, the coordinates at the global system (*V*_*g*_) can be computed with ${M}_{i}^{{\prime} }s$ and local coordinates *V*_*l*_. Similarly, different mocap devices also introduce various coordination systems. For example, the Kinect defines the origin of the coordination system at the center of its infrared sensor. X grows to the sensor’s left; Y grows up to the sensor’s tilt; Z grows out in the direction the sensor is facing. The values of x, y, and z can be negative or positive and depend on the relative locations of the sensing subjects to the sensor. Most datasets with Kinect technologies directly have their skeleton joint positions under the device coordinate system. By implementing the vectorized transformation process shown in Fig. 4, these datasets can be translated to the subject coordinate system or the global coordinate system. In addition, depending on the definition of the x, y, and z orientations, the cartesian system may have different setups, such as XYZ, YXZ, or ZYX. Through multiplying proper rotation matrices, all coordination systems can be aligned as XYZ setups for the ease of data processing in the future.

#### Data segmentation

Each dataset has its unique separation of activities and one obvious consequence is the length of samples is different, even in the same dataset. Many quantitative implementations require the input data have the same length or size for ease of practical use. Therefore, the CML dataset needs proper data segmentation. After resampling, this study utilized a sliding window to align and count the length of all samples in the datasets. The average frame length of all construction-related activities was counted for the in-lab experiment dataset (around 92 to 96.9 frames). Then a standardized frame number, such as 100 and 120, was chosen for different activities. However, people may perform the same activity at a different speed. Therefore, the standardized frame number needs to cover at least 80% of activity postures. If the activity finishes early, the rest of the frames will remain the same as the last motion frame. Following this principle, the proper frame number for each activity was selected and the data segmentation was manually conducted with the proper frame length.

#### Data format of the cml dataset

To allow efficient data query and easier data sharing, this study chose to export the CML Dataset as JavaScript Object Notation (JSON) files. JSON is a lightweight data format that can be directly accessed online and stored and queried with NoSQL databases, such as MongoDB. It can be conveniently accessed and imported with major computational and programming tools and converted to other conventional data formats, such as ASF/AMC, c3d, BVH, TXT, and CSV.

### Dataset annotation

The conventional rule to group human activities is based on their complexity. For example, Aggarwal and Ryoo categorized activities as gestures, actions, interactions, and group activities^41^. Gestures are elementary movements of a person’s body part, which label a person’s activities as “rotating two arms,” “raising a leg,” etc. Activities are continuous motions that are composed of multiple postures, such as “catching,” “pulling,” and “carrying.” Interactions are activities involving one person and another person or a person and an object. Unsafe behaviors are the major cause (over 80%) of accidents^42^ and quantitative skeleton postures can be used for activity recognition^43,44^. Another career health factor, which is closely related to WMSDs is long-time awkward posture during task execution^45^. For regular working activities, researchers highlighted the workers’ activities as “productive mode”, “semi-productive mode”, and” non-productive mode”^46,47^. Therefore, all activities in our dataset are divided into two broader groups, construction-related activities (production activities, unsafe activities, and awkward activities) and non-construction-related activities (common activities and other activities). Production activities are directly related to construction tasks and unique activities related to the industry. They could be used to identify workers’ working status and efficiency. Unsafe activities are activities that expose workers to high risks and potentially cause accidents. Awkward activities may not be directly related to accidents, but they are associated with long-term, work-related musculoskeletal disorders (WMSDs), which are harmful to the long-term health of workers. Common activities are general activities that are similar to activities in daily life, such as “sitting,” “standing,” and “walking.” To annotate the samples in the dataset, this study captured the three types of predefined construction activities. The labels are typical construction activities and whether the label belongs to unsafe or awkward activities were determined by existing ergonomic theories and models, for example, the rapid entire body assessment (REBA)^48^, the Rapid Upper Limb Assessment (RULA)^49^, Ovako Working Posture Assessment System (OWAS)^50^, the Manual Handling Assessment Chart (MAC)^51^, Posture, Activity, Tools and Handling (PATH)^52^, Washington State’s ergonomics rule (WAC 296-62-051)^53^.

#### Production activities

Much existing research has identified the major production activities and introduced the concept of using them to define and measure productivity in construction projects^54^. Also, production activities can be used to evaluate the working state of workers^55^. However, some activities have similar posture sequences as nonproduction activities. For example, “moving with hands empty” and “transporting rebar” may have similar skeleton motions. Therefore, to ensure proper labeling, this study only labeled activities as production activities when they had clear relevance to building materials, tools, or construction equipment, like “welding,” “drilling,” “nailing,” “bolting,” and “sawing.”

#### Unsafe activities

Many studies have reported that 80–90% of accidents are associated with workers’ unsafe activities^56,57^. The Occupational Safety and Health Administration (OSHA) defined the most critical unsafe activities based on reported accident statistics^58^. Extending OSHA’s statistics and reports, many researchers have clearly defined typical unsafe behaviors. For example, Han and Lee^59^ derived six construction crew unsafe activity categories, including falls, transportation, contact with objects and equipment, exposure to harmful substances/environments, assaults and violent acts, fires, and explosions. Hinze *et al*.^60^ attributed accidents to 20 possible unsafe activities, including falls from elevation, falls from ground level, electrocution (power lines), electrocution (building power), electrocution (faulty facility wiring), electrocution (faulty construction tool/wiring), electrocution (other), struck by equipment, struck by falling material, struck by material (other than falling material), caught in/between equipment, caught in/between material, cave-in, explosion, fire, explosion/fire, asphyxiation, drowning, natural causes, and other. Choudhry *et al*.^61^ reported that a lack of any of five construction resources may result in unsafe activities for the construction crew. These resources include personal protective equipment, housekeeping, access to heights, plant and equipment, and scaffolding. Based on these studies, this study labels unsafe behavior when relevant to the following activities or events: (1) falling from different levels or ladders, including slipping, tripping, climbing/jumping ladders/stairs, and reaching; (2) taking off personal protective equipment, including hard hats, gloves, vests, shoes, and glasses; (3) being close to fires and explosions, including smoking; (4) being exposed to assaults and violent acts, including hitting, kicking, and beating. Finally, 36 unsafe activities were identified and used to annotate the final CML Dataset.

#### Awkward activities

Musculoskeletal disorders account for 33% of all newly reported occupational illnesses and 77% of those of construction workers, making them the single largest cause of work-related illness^62^. As the major cause of these disorders, this study lists a separate awkward activity category. Based on the ergonomic analysis, many researchers have proposed quantitative definitions of the awkward activities of construction workers. For example, Jaffar *et al*.^63^ proposed seven generic awkward postures and activities, including leaning sideways, bending down, reaching overhead, flaring the elbows out to the side, bending the wrist, bending the neck down, and twisting part of the body. James *et al*.^64^ suggested postures that deviate from the neutral position, such as gripping, kneeling, lifting, bending, working overhead, twisting, using vibrating equipment, squatting, and overreaching. Based on these studies, this study developed 11 awkward activity labels.

#### Common activities

Besides the above construction-related activities, generic activities, such as walking, sitting, and standing, are categorized as common activities. These activities are supplementary to or part of more complicated activities.

#### Other activities

In addition to the four major types of activities related to construction, irrelevant activities are labelled as “other,” such as “moonwalking” or “applying cream to the face.”

### Protocol of manual annotation

Due to distinctive experiment designs, the descriptive labels for the same activities may be different. For example, the activity of jogging in the MSRAction3D Dataset was labelled as “jogging,” but in the UTD-MHAD3 Dataset, it is “jogging in place.” The labels “approaching” and “departing” in the SBU Kinect Interaction Dataset have a walking direction due to the experiment design. Therefore, to ensure the consistency of all labels, the manual annotation of the CML Dataset follows three rules.

- **Simple and representative**. The name of the label should be simple, short, and representative of the nature of an activity. For example, “jogging in place” and “jogging slowly” will be labelled as “jogging”; “walking forward” and “departing” will be labelled as “walking.”
- **Nondirectional**. As the coordination systems of all datasets are translated into the subject’s coordinates, the directional labels will be merged as the same label. For example, “walking to east” and “walking forward” will be labelled as “walking.”
- **Clear and conservative**. To avoid potential mistakes, ambiguous activity data samples will not be labelled and will be removed.

As shown in Table 2, all labels are predesigned activities based on the literature review. All dataset are publicly accessible and useable with proper citation. For the Licenses listed as “--”in table, the dataset owner does not specify the type of licenses. The in-lab experiment collected the standard posture frames for all construction-related activities. The samples from the public dataset were compared with all standard activities and annotated as the one that has the highest similarity (lowest differences in skeleton joint movements). When two activities are similar to each other, the “label” in the categories of construction-related activities and unsafe activities has a higher priority. Table 3 shows a sample of merged labels.

**Table 2:** A Label Sample of the Manual Annotation.

	Dataset Name	License	URL
0	In-lab Experiment	Own	
1	CMU Mocap	Permitted	http://mocap.cs.cmu.edu/
2	HMD05 Mocap	CC BY SA 3.0	http://resources.mpi-inf.mpg.de/HDM05/
3	Berkeley MHAD	BSD-2	https://tele-immersion.citris-uc.org/berkeley_mhad
4	NTU+RGBD 120	Not Permitted	https://rose1.ntu.edu.sg/dataset/actionRecognition/
5	SBU Kinect Interaction Dataset	ODbL	https://www.kaggle.com/datasets/dasmehdixtr/two-person-interaction-kinect-dataset
6	MSR Action3D Dataset	--	https://sites.google.com/view/wanqingli/data-sets/msr-action3d
7	CAD60 Dataset	CC BY 4.0	http://pr.cs.cornell.edu/humanactivities/data.php
8	UTKinect-Action3D Dataset	--	http://cvrc.ece.utexas.edu/KinectDatasets/HOJ3D.html
9	UCF Dataset	--	http://www.syedzainmasood.com/research.html
10	Microsoft Research Cambridge-12	--	https://www.microsoft.com/en-us/download/details.aspx?id=52283
11	Human 3.6	--	http://vision.imar.ro/human3.6m/description.php
12	PKU_MMD	--	https://www.icst.pku.edu.cn/struct/Projects/PKUMMD.html?aimglfkfkfcjmopp
13	SYSU 3D	--	https://www.isee-ai.cn/~hujianfang/ProjectJOULE.html
14	UTD Multimodal Human Action Dataset (UTD MHAD1)	--	https://personal.utdallas.edu/~kehtar/UTD-MHAD.html
15	UTD MHAD2	--	https://personal.utdallas.edu/~kehtar/UTD-MHAD.html
16	UTD MHAD3	--	https://personal.utdallas.edu/~kehtar/UTD-MHAD.html

**Table 3:** Statistics of the CML Dataset.

Activity label	No. Trail	Original label in the source datasets
“Jogging”	62	MSR jogging; UTD-MHAD3 jogging in place
“Walking”	5,416	CMU backward; CMU sideways; CMU walk; CMU walk with arms out; HDM05 deposit; HDM05 walk; NTU+RGBD 120 step on foot; NTU+RGBD 120 walking apart; NTU+RGBD 120 walking towards; SBU approaching; SBU departing; UTKA walk; UTD-MHAD3 forward lunge (left foot forward); UTD-MHAD3 walking in place

## Data Records

The final CML dataset has been stored in the FigShare repository^65^. The relevant public datasets’ doi can be found in Table 1 and their URLs can be found in Table 2. Table 4, Table 5, and Table 6 list the data statistics and data structures of the dataset.

**Table 4:** Data Structure of the JSON Dataset.

	All activities	Other activities	Construction-related activities			
			Unsafe activities	Awkward activities	Production activities	Common activities
Number of labels	225	152	38	10	12	13
Number of samples	146,480	85,205	36,778	5,101	5,105	14,291
File size	23.93 GB	13.40 GB	5.98 GB	0.69 GB	0.72 GB	3.14 GB

**Table 5:** The Labels of Each Activity Category.

Key	Data Type	Sample Data	Description
“data source”	String	“CMU”	Source of the dataset
“original label”	String	“back somersault”	Original label of the raw dataset
“source file”	String	“88_01_01.txt”	The filename of the original raw dataset
“label”	String	“kicking”	New label in the integrated dataset
“frames”	Number	101	Total number of frames in one data sample
“coordinates”	Enumerate	“x y z”	The coordination system of the data sample
	<String>		
“activity type”	Enumerate	0	“0” – other activities; “1” – unsafe activities; “2” – awkward activities; “3” – production activities; “4” – common activities
	<Number>		
“joints”	Enumerate	15	“15” – 15-joint skeleton model; “20” – 20-joint skeleton model
	<Number>		
“calibrated”	Boolean	TRUE	Indicates whether joints data are interpolated and computed
“bones”	Array	[‘Head’, …,]	The list of joints/bones
“tdata”	Object	{“Frame_ID”: [Data…], …}	An object with each frame and its corresponding data array
“bdata”	Object	{“Bone_name”: [Data…], …}	An object with each joint and its corresponding temporal data array

**Table 6:** The Structure of Four Deep Learning Models.

Category	Activity Type	Labels
Unsafe Activities	1	"back pain": 0, "beating": 1, "calling phone": 2, "chest pain": 3, "climb down": 4, "climb up": 5, "cough": 6, "dialing phone": 7, "dropping": 8, "ducking": 9, "falling": 10, "hanging": 11, "headache": 12, "hitting": 13, "jogging": 14, "jumping": 15, "kicking": 16, "lean back": 17, "neck pain": 18, "pick up and throw": 19, "punching": 20, "running": 21, "smoking": 22, "staggering": 23, "stair down": 24, "stair up": 25, "step over": 26, "swing": 27, "take off a hat and cap": 28, "take off a shoe": 29, "take off glasses": 30, "take off jacket": 31, "throwing": 32, "vomiting": 33, "walk on uneven terrain": 34, "wrestle": 35
Awkward Activities	2	"arm curl": 0, "bending": 1, "carrying": 2, "crawling": 3, "elbow to knee": 4, "hand catch": 5, "pulling": 6, "pushing": 7, "squats": 8, "twists": 9, "weight lifting": 10
Production Activities	3	"bolt tightening or loosening": 0, "coiling a rope": 1, "cutting nails": 2, "digging": 3, "drawing": 4, "driving": 5, "dynamic calibration": 6, "exchanging objects": 7, "hammer": 8, "knocking": 9, "moving object": 10, "poking ground": 11, "sawing": 12
Common Activities	4	"get up from floor": 0, "lay down": 1, "picking up": 2, "put on a hat and cap": 3, "put on a shoe": 4, "sit down and stand up": 5, "sitting": 6, "stand up": 7, "standing": 8, "turning": 9, "walking": 10, "wearing clothes": 11, "wearing glasses": 12
Other Activities	0	“apply cream on face”, “apply cream on hand”, “backflip”, “ball up paper”, “baseball action”, “baseball swing”, “basketball dribbling”, “basketball shooting”, “basketball signals”, “blow nose”, “bowing”, “bowling”, “brushing hair”, “brushing teeth”, “capitulate”, “carry suitcase”, “cartwheel”, “change weapon”, “checking time”, “cheer up”, “chopping wood”, “clap above head”, “clapping”, “close a box”, “closing umbrella”, “cooking”, “counting money”, “cross arms on the chest”, “cross hands in front”, “cross toe touch”, “curtsy”, “cutting paper”, “dance”, “direct traffic”, “drinking water”, “eating”, “fan self”, “fishing”, “fold paper”, “golf swing”, “greeting”, “had enough”, “hand signals”, “hanging a picture”, “high five”, “hit head”, “holding a baby”, “hopping”, “hugging”, “hushing”, “imitating animals”, “jumping jack”, “laugh”, “lift open window”, “look around”, “make OK sign”, “make victory sign”, “making dough”, “marching”, “mixing water”, “moonwalk”, “mopping”, “nod head and bow”, “nursery rhyme”, “open a box”, “open bottle”, “opening pill container”, “opening umbrella”, “pat on back”, “placing golf ball”, “placing golf tee”, “planting a flower”, “play magic cube”, “playing drums”, “playing piano”, “playing violin”, “pointing finger”, “posing”, “pouring”, “purchases”, “put object into bag”, “put on bag”, “put on headphones”, “put palms together”, “put something inside pocket”, “puts hands on B’s shoulders”, “reading”, “relaxing”, “rinsing mouth with water”, “rock-paper-scissors”, “rotate arms”, “rub shoulder”, “rub two hands”, “salute”, “sewing”, “shake head”, “shaking hands”, “shaving”, “shelter someone from harm”, “shooting”, “skateboard”, “skier”, “snap fingers”, “sneaking”, “sniff and smell”, “soccer”, “spin”, “spray deodorant”, “stand mixing batter”, “stand slicing object”, “staple book”, “start system”, “story”, “stretches”, “support somebody”, “sweeping”, “swimming”, “swordplay”, “T-Pose”, “table tennis”, “tai chi”, “take object out of bag”, “take off bag”, “take off headphones”, “take out something from pocket”, “taking a selfie”, “taking photo”, “tear up paper”, “tennis serve”, “tennis swing”, “throw and catch ball”, “thumb down”, “thumb up”, “toss a coin”, “vacuuming”, “vault”, “waiting”, “walking dog”, “wash self”, “wash windows”, “waving”, “wearing contact lenses”, “whisper”, “wield knife”, “wind it up”, “wipe floor”, “wiping face”, “working on computer”, “writing”, “writing on whiteboard”, “yawn”, “yoga”

**Fig. 5:**
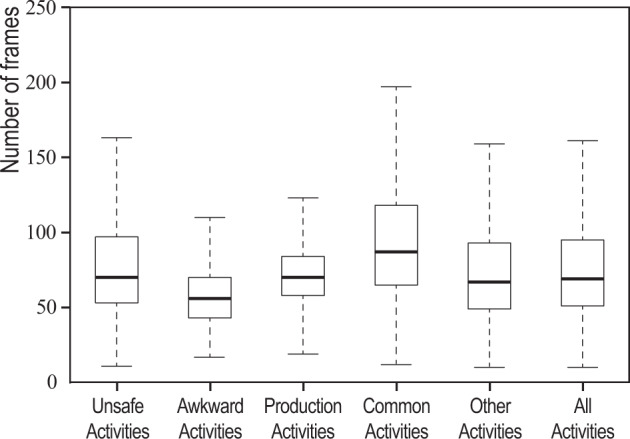
Frame number statistics of samples in each activity category.

Table 4 summarizes the statistics of the final CML Dataset. In total, there were 146,480 samples extracted from the original public datasets and in-lab experiment datasets. Originally, there were 225 classes of activities labelled for all activities. After aggregation, only 73 labels were regarded as related to construction activities, and only 61,275 samples were suitable for future development. The size of all JSON files in the CML Dataset was more than 10 gigabytes. Figure 5 shows the boxplot of the frame numbers of all activities. The average frame number for construction-related activities and all activities were 92 and 96.9. Due to the public dataset license requirements, this study only shared the processed data sample from datasets that allow redistribution and sharing. In summary, the shared dataset has 6,131 samples (among them, 4,333 samples are construction-related activities). Since the rest of the datasets are publicly available, this study provides a code repository that allows users to construct the complete datasets with a bvh parser and the skeleton converter.

The CML data format for storage is JSON for easier importing, assessing, and sharing. The file can be divided into two parts. The first “meta-data” part only stores the information related to the data summary, the original dataset source, and the joint structure and includes all the keys except “tdata” and “bdata.” The second “formal-data” part only includes “tdata” (an object encloses frames of all joints over time) and “bdata” (an object includes time-series data of each joint). All “formal-data” is stored at a standardized frame rate/sampling rate of 30 Hz. The detailed data structure is listed in Table 5.

Table 6 shows all labels that are annotated in the CML Dataset. The first four categories are construction-related activities, and the last category is irrelevant activities. To maintain the completeness of the dataset, the “other activities” are kept in the CML Dataset, but for future development, researchers can only use the first four activity categories.

## Technical Validation

### Dataset samples for testing

This section intends to validate the developed dataset and ensure its usability and reliability in practice. This study selected five widely accepted deep learning models to examine the performance of the developed CML Dataset. By doing so, it can demonstrate how the unified datasets can be easily used as the standardized inputs for complex networks. Also, the training and recognition results are comparable to other datasets and can be found tuned by other pre-trained models. In addition, the validation results can clearly show the trade-off between computational cost and recognition accuracy of using the CML dataset.

The selected algorithms include *(1) long short-term memory (LSTM), (2) bidirectional LSTM networks*^66^*, (3) LeNet-5*^67^*, (4) AlexNet*^68^*, and (5) ResNet-50*^69^. LSTM is the most widely used recurrent neural network that models the temporal and context relationships of input data by implementing an additional forgetting gate and internal memory cell. Bidirectional LSTM networks extend the LSTM model by introducing bi-directional relationships among samples. In this study, the bidirectional LSTM network is stacked with two layers with 90 neurons and a 0.5 dropout rate, followed by two fully connected layers with the activation function ReLU^70^. LeNet-5 is a classical convolutional neural network (CNN) but incorporates spatial relationships among high-dimensional data. AlexNet extends LeNet-5 and shows higher accuracies on large-scale image and video datasets. ResNet-50 is a residual network and has an additional identity mapping capability. The detailed network structures and parameters of the all five testing algorisms are summarized in Table 7. The input sizes are different for the 15-joint and 20-joint systems. For example, for AlexNet, the sizes are 3 by 100 by 90 and 3 by 100 by 120 for the 15-joint and 20-joint systems, respectively.

**Table 7:** Sample Sizes of the Validation Tests.

Layer	Model			
	Bi-LSTM LSTM	LeNet-5	AlexNet	ResNet-50
Input*	(1, 100, 45)	(1, 100, 45)	(3, 100, 90)	(3, 100, 90)
	(1, 100, 60)	(1, 100, 60)	(3, 100, 120)	(3, 100, 120)
1	LSTM layer	Conv2d:	[11 × 11, 64] × 2/4	7 × 7, 64, stride 2
		5 × 6 × 6	Max pooling: 3 × 3/2	Pooling: 3 × 3, stride 2
2	Dropout layer	Pooling:	[3 × 3,192] × 2	[1 × 1, 64; 3 × 3, 64; 1 × 1, 256] × 3
		2 × 2/2	Max pooling: 2 × 2	
3	LSTM layer	Conv2d:	[3 × 3,384]	[1 × 1, 128; 3 × 3, 128; 1 × 1, 512] × 4
		5 × 6 × 16		
4	Dropout layer	Pooling:	[3 × 3,256]	[1 × 1, 256; 3 × 3, 256; 1 × 1, 1024] × 6
		2 × 2/2		
5	Full connection	Full connection	[3 × 3,256]	[1 × 1, 512; 3 × 3, 512; 1 × 1, 2048] × 3
			Max pooling: 3 × 3/2	Average pooling: 7 × 7
6	Full connection	Full connection	Full connection × 3	Full connection

Due to the complexity of activities and individual differences in experiment subjects, the sequence length *T* of each activity sample may differ. This study utilized the sparse sampling strategy to unify the sequence length *T* for different full-length, ensuring all samples can be fed into networks with the same dimensions. For CNN-based networks, the body skeleton is ${x}_{t}\in {R}^{3\times N}$, where *N* represents the number of joints and 3 is the dimension of coordinates. At each time step *t* ∈ *T*, *t* is the index of frames. Therefore, the input for CNN-based networks is the skeleton sequences $X\in {R}^{3\times N\times T}$, and for LSTM-based networks, the input size is $X\in {R}^{3N\times T}$.

The dataset was tested with the different number of iterations and proportion/size of the dataset. The iteration test was designed to examine the efficiency of using the CML Dataset to achieve a sufficiently accurate model. The different sample size test intends to examine if there is sufficient data quantity and efficient data size to reach an acceptable accuracy. The examined sample sizes are listed in Table 8.

**Table 8:** Recognition Performance of Algorithms Over Epochs.

	Sample 1	Sample 2	Sample 3	Sample 4	Sample 5	Sample 6
Proportion of total samples	10%	30%	50%	70%	90%	100%
Total samples	6,128	18,382	30,637	42,893	55,147	61,275
Training samples	3,676	11,029	18,382	25,735	33,088	36,723
Validation samples	2,452	7,353	12,255	17,158	22,059	24,552

All algorithms were programmed with Python language with the Pytorch package. The testing desktop computer was configured with Intel i7-11700@ 2.50GHz CPU (8 core, 16 threads) and GeForce GTX 3060Ti GPU. Based on the sparse sampling strategy, each training batch had 256–1024 sequences, and the total training epoch was 10,000. The learning rate was set as 0.00001, and the Adaptive Moment Estimation (ADAM) algorithm^71^ with a decay rate of 0.001 was adapted to optimize the cross-entropy loss.

### Validation results

**Fig. 6:**
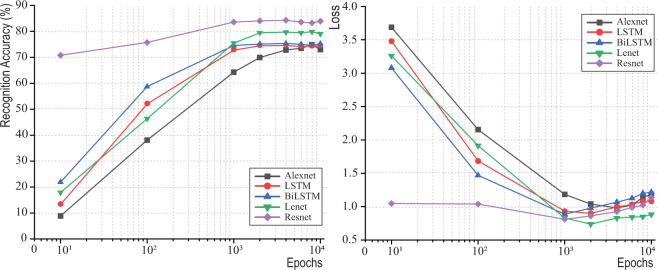
Different deep-learning models’ recognition accuracies and losses over epochs.

Figure 6 and Table 9 show the performance of five deep learning models’ accuracy of development over epochs. Most algorithms convert at 1,000 epochs. The final 10,000-epoch run only slightly increased the recognition accuracy. When the epoch number is more than 4,000, the loss begins to increase, and the recognition accuracy becomes fluctuated. Therefore, the developed CML Dataset is able to generate acceptable and usable learning models quickly at 1,000 epoch runs. The average model training time is less than one hour.

**Table 9:** Recognition Performance of Algorithms Over Epochs.

Epochs	Recognition Accuracy				
	LSTM	Bi-LSTM	LeNet-5	Alex Net	Resnet
10	13.48%	21.91%	17.94%	8.85%	70.81%
100	52.06%	58.72%	46.36%	38.08%	75.76%
1,000	73.00%	74.58%	75.46%	64.31%	83.56%
2,000	74.67%	75.12%	79.45%	69.92%	84.06%
4,000	74.77%	75.36%	79.73%	72.82%	84.32%
6,000	74.43%	75.00%	79.44%	73.50%	83.56%
8,000	74.46%	74.64%	79.86%	74.87%	83.26%
10,000	74.62%	75.24%	79.04%	72.99%	83.92%

**Fig. 7:**
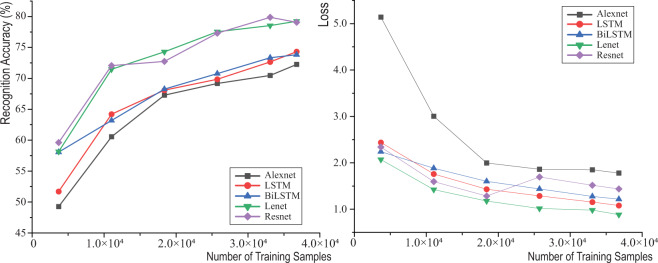
Different deep-learning models’ recognition accuracies and losses with different sample sizes.

Figure 7 shows the recognition accuracies and losses with different sample sizes. The accuracies and losses were averaged from multiple shuffled training tests. The training dataset used only a proportion of the whole CML Dataset as shown in Table 8. The testing samples were unused samples, so smaller training samples had larger testing samples. The aim of this test was to ensure the sufficiency of data for activity recognition. Due to the large and changing testing sample size, the loss of a model indicates the data sufficiency when the sample size was relatively small. As can be seen, the loss converges around 25,000 samples, suggesting the developed CML Dataset is sufficient to train a reliable activity recognition model.

Conventional activity recognition methods classify construction activities into few types. For example, Gong *et al*.^17^ used characterized images to classify construction activity into five categories traveling, transporting, bending down, aligning, and nailing. Escorcia *et al*.^72^ used bag-of-poses represented color and depth data from Kinect sensor to recognize 5 types of drywall construction activities. Yang *et al*.^19^ proposed a dense trajectories representation method to recognize 11 common construction activities from videos. Recently, Akhavian and Behzadan^24^ compared various classification algorithms and reported that neural network models provide higher accuracy and allow more types of activities to be recognized. However, neural network models, especially deep learning models, highly rely on datasets that have a large quantity of data and comprehensive labels. The CML dataset developed by this study not only composed a dataset with 60 construction-related activity labels but also constructed a standardized large-scale dataset based on public datasets with more than 100 non-construction-related activities. Such enrichment in the data source can greatly improve the performance of activity recognition models and can be expanded to other usage domains. In the technical validation case, the average action recognition accuracy of the five algorithms obtained an accuracy of 74.62 to 83.92%. Given the large number of different labels, the CML dataset can make a significant contribution to the industry.

## Usage Notes

Accurate motion recognition algorithms depend on reliable and ample datasets. Although activity recognition is vital to managing construction tasks and avoiding injuries, a dataset specifically designed for the construction industry is still lacking. At the same time, modern machine learning models have high requirements for the quality and quantity of datasets; accessible and lightweight is the premise of implementing artificial intelligence in a specific industry. The CML Dataset was developed to serve the above purposes and provide a data infrastructure for the development of sophisticated models and tools. All data has been properly aligned and cleaned for ready usage and was stored in the most compatible format. All relevant labels have been manually validated and annotated to ensure their correctness. The validation tests suggest the developed CML Dataset is sufficiently large and rich to train accurate and agile learning models. It is also versatile enough to be implemented in both vision-based and IMU-based motion capturing systems with different devices and equipment.

The developed CML Dataset can be used in the development of safety and productivity assessment models and toolsets. Through recognizing production activities, the task load and its physical demands and human worker capacities can be assessed. This can be utilized in productivity computation and in organizing construction schedules. Given the unsafe and awkward activities recognition, project management platforms can provide early warnings and proper training programs to construction workforces. Also, the intensity of awkward activities can be used to evaluate long-term chronic harm to workers who potentially suffer from WMSDs. In addition, activity recognition can be extended to coordinate human-robot collaboration in the future. Therefore, the CML Dataset plays an essential role in sustaining these applications and the development of the construction industry.

The CML Dataset mingled both vision-based and IMU-based mocap datasets to assure its generality and versatility. However, the joints represented by both systems are not perfectly aligned; for example, vision-based systems tend to predict the skeleton joints as the geometric center of body parts, but IMU-based systems recorded joint movements at the point where the sensors were attached. This results in some slight inconsistency in postures. In addition, construction activities are highly tool-dependent, which may result in different activities but the same skeleton movements. Therefore, the motion labels annotated by this study are generic and imprecise. High-resolution activity recognition requires inputs from other sensing sources.

The CML Dataset has predefined 60 labels for construction-related activities, but there are many more different types of activities in practice. Therefore, the 60 labels are designed to be generic to encompass similar activities. Given the complexity and variety in construction tasks and process organization to further differentiate more professional activities, additional information (such as tools and equipment used by workers, working environment, site context, etc.) is necessary.

## Data Availability

This study utilized Mathwork Matlab 2020a to parse and export the ASF/AMC and BVH files. The open-source code used for parsing these files can be obtained from https://github.com/lawrennd/mocap. This study utilized Python 3.7.6 and extended a 17-joint BVH conversion package, video-to-pose3D (https://github.com/HW140701/VideoTo3dPoseAndBvh), to generate BVH files. The newly developed package can transform 15 or 20-joint models’ JSON files into BVH files. The developed code can be accessed with the following URL: https://github.com/YUANYUAN2222/GIT_json_to_BVH. Meanwhile, the code could be used to retag and process different datasets (i.e., Resampling and Skeletal structure alignment) is made public on the GitHub (https://github.com/YUANYUAN2222/Integrated-public-3D-skeleton-form-CML-library), which allow all readers and potential users to process the source dataset by themselves.
